# Inattention and task switching performance: the role of predictability, working memory load and goal neglect

**DOI:** 10.1007/s00426-019-01214-1

**Published:** 2019-06-27

**Authors:** Gizem Arabacı, Benjamin A. Parris

**Affiliations:** Department of Psychology, Bournemouth University, Talbot Campus, Fern Barrow, Poole, Dorset BH12 5BB UK

## Abstract

Inattention is a symptom of many clinical disorders including attention deficit hyperactivity disorder (ADHD) and is thought to be primarily related to limitations in working memory. In two studies, we investigated the implications of inattention for task switching performance. In study one, we measured task switching performance using predictable and unpredictable conditions in adults who self-rated inattention and other ADHD-related tendencies. Tasks required proactive control and reactive control, respectively, under both high and low working memory loads. Results revealed that inattentive, but not hyperactive/impulsive traits, predicted switch costs when switching was predictable and working memory load was high. None of the ADHD traits were related to unpredictable switch costs. Study two was designed to: (1) de-confound the role of proactive control and the need to keep track of task order in the predictable task switching paradigm; (2) investigate whether goal neglect, an impairment related to working memory, could explain the relationship between inattention and predictable task switching. Results revealed that neither predictability nor the need to keep track of the task order led to the association between switch costs and inattention, but instead it was the tendency for those high in inattention to neglect preparatory proactive control, especially when reactive control options were available.

## Introduction

Inattention is a symptom of many clinical and mental disorders although it is most closely associated with attention deficit hyperactivity disorder (ADHD). ADHD manifests itself in three presentations: predominantly inattentive (ADHD-I), predominantly hyperactive/impulsive (ADHD-HI) and combined (ADHD-C: American Psychiatric Association, 2013). Our present focus is on the symptom of inattention as characterised as a difficulty in sustaining attention, listening/following conversations/instructions, and organising. Furthermore, it is associated with mind wandering-like experiences (e.g. “mind seems elsewhere” or “distractible by unrelated thoughts”), forgetfulness and hesitation to engage in activities requiring sustained mental effort (APA, 2013).

Whilst ADHD is a widely diagnosed neurodevelopmental disorder with prevalence rates of 5–10% in childhood and 4.4% in adulthood (Kessler et al. 2005), research has suggested that inattention, like the other ADHD symptoms, is best thought of as being on a continuum as opposed to being categorically different from sub-clinical levels of the disorder (Barkley & Murphy, 1998). Based on this view, tendencies of inattention, hyperactivity and impulsivity are also experienced by sub-clinical populations, and, those with a clinical diagnosis represent the extreme end of the spectrum. Measuring ADHD-related experiences on a continuous scale has been supported by taxometric studies (Haslam et al. 2006; Salum et al. 2014) and studies report a significant impact of ADHD-related traits at sub-clinical levels (Elisa, Balaguer-Ballester, & Parris, 2016; Overbey, Snell, & Callis, 2011; Seli, Smallwood, Cheyne, & Smilek, 2015).

Diamond (2005) argued, and research has supported, that inattention is primarily related to limitations in working memory (Nigg, 2006; Barkley, 1994; Nigg, 2001). For example, Martinussen et al. (2005) revealed that inattentive (but not hyperactive/impulsive) symptoms were related to verbal and visuospatial working memory (WM) impairments in clinical samples (see also Klingberg et al., 2005). Using non-clinical samples, Gathercole et al. (2008) revealed that low WM capacity children had more inattentive traits than a high WM group. Lui and Tannock (2007) also reported that poor performance on WM tasks predicted parent-rated inattentive traits at sub-clinical levels. In adults, Elisa et al. (2016) found that self-reported inattentive traits predicted the performance on verbal WM at sub-clinical levels. Thus, the literature provides evidence for a link between inattentive symptoms and WM performance at clinical and sub-clinical levels.

Although the core executive functions of WM, response inhibition and task switching are thought to be independent processes research also suggests that they may still be interrelated (Friedman & Miyake, 2017). Therefore, WM limitations in inattentive individuals might produce impaired performance on other executive function tasks, such as task switching (e.g. Emerson & Miyake, 2003; Liefooghe, Vandierendonck, Muyllaert, Verbruggen, & Vanneste, 2005; Miyake, Emerson, Padilla, & Ahn, 2004). Moreover, since the relationship between inattention and task switching performance has not yet been investigated it is possible that inattention also leads to impaired task switching performance irrespective of WM involvement.

Task switching paradigms (TSPs) measure the cognitive flexibility required to achieve task goals when the environment is constantly changing. Switching refers to an individual’s ability to self-adjust their performance based on the current requirements to achieve task goals. To perform a switch, attentional resources must shift to the relevant task set (Allport, Styles, & Hsieh, 1994; Jersild, 1927; Rogers & Monsell, 1995; Spector & Biederman, 1976). Task-set refers to the parameters required to perform a particular task such as stimulus identification, response selection and response execution (Logan & Gordon, 2001; Vandierendonck, Liefooghe, & Verbruggen, 2010).

In TSPs, participants are often asked to perform two tasks in quick succession. A participant might be required to repeat the same task (e.g. judging whether a number is higher or lower than 5) a number of times before they are asked to perform another task (e.g. judging whether a presented number is odd or even). These two tasks would be presented such that there are a number of repeat and switch trials in each experiment. Switching from one task to another is associated with longer reaction times (RTs) and higher error rates compared to repeat trials. These performance costs are referred to as *switch costs* (Meiran, Chorev, & Sapir, 2000; Altmann, 2004; Dreisbach, Haider, & Kluwe, 2002; Koch, 2001; Meiran, 1996; Rogers & Monsell, 1995). Various accounts have been made to explain the source of switch costs including the role of interference from the previous task-set (Allport et al., 1994) and task-set reconfiguration (Rogers & Monsell, 1995).

It has been argued that task switching performance calls upon WM processing for the activation and maintenance of task-sets (Emerson & Miyake, 2003; Liefooghe et al., 2005; Miyake et al., 2004) and for tracking sequential action plans (Bryck & Mayr, 2008). However, research has failed to find an effect of WM on task switching (Kane, Conway, Hambrick, & Engle, 2007; Logan, 2004 but see Liefooghe, Barrouillet, Vandierendonck, & Camos, 2008). Nevertheless, the reliance of task switching on WM might depend on the parameters of the task switching requirements. For example, WM would be required to prepare for the upcoming task when the information about the upcoming task is available in advance as in predictable TSPs. The reconfiguration account of task switching suggests that switching requires a mental form of ‘gear changing’ (*task*-*set reconfiguration*) to trigger the task-specific processes, such as retrieving the relevant *task*-*set*. If advanced knowledge and sufficient time is allowed, individuals are able to prepare for the upcoming task, thereby reducing the switch cost (reconfiguration view; Rogers & Monsell, 1995). This has been referred to as *proactive control* (Braver, Gray, & Burgess, 2007).

The distinction between proactive and reactive control was first introduced by Braver et al. (2007) who proposed the dual-mechanism theory of cognitive control, suggesting two types of control for the flexible, goal-related behaviour. Proactive control refers to the active maintenance of information (e.g. general task instructions, relevant information from the previous stimuli or salient cues) that is beneficial for responding to upcoming stimuli (Engle & Kane, 2003; Kane et al., 2007), and, the ability to make use of the previous stimuli to predict the upcoming event (Braver et al., 2007). Reactive control involves retrieving contextual information that is relevant for current decision making. Proactive control requires maintaining previous knowledge and using this to respond efficiently when a future event is consistent with expectations. In an attempt to integrate the role of preparation and interference during switching, Vandierendonck et al. (2010) evaluated the switch costs in two processing stages: preparation and stimulus-based processing. They suggested that two forms of control are needed: reactive control to overcome the interference due to task-set inertia and proactive control to shield the task relevant goal and instructions. Research has found that high WM capacity participants are better at making use of prior information or cue information to predict the upcoming event in various tasks. High WM capacity participants are also more able to maintain the goal relevant information in memory (Engle & Kane, 2003; Unsworth, Schrock, & Engle, 2004; Redick, Calvo, Gay, & Engle, 2011) and use this information proactively to bias their responses (Redick et al., 2011). In individuals reporting high levels of inattention one would, therefore, be expected to exhibit poorer use of cue information and poorer maintenance of goal relevant information. In sum, during predictable task switching, a form of proactive control is needed to perform advanced reconfiguration to prepare for the upcoming stimuli and shield the relevant goal, and this is related to WM capacity. When the use of proactive control is not possible (i.e. future events cannot be reliably predicted), individuals rely on reactive control (Redick, 2014). Furthermore, reactive control would be used to overcome task set inertia.

## Task switching and ADHD

Whilst there has been no research, to our knowledge, considering task switching performance and inattention, studies using TSPs on participants with ADHD have revealed conflicting results. Using the same task, Cepeda et al. (2000) and Kramer et al. (2001) reported larger switch costs in those with ADHD compared to controls while Oades and Christiansen (2008) failed to find a significant difference in switch costs. Other studies reported significantly larger switch costs for ADHD participants (King, Colla, Brass, Heuser, & von Cramon, 2007) while others did not (Rauch, Gold, & Schmitt, 2012). For example, Wu et al. (2006) investigated the switching performance for those with and without ADHD under WM load. Participants were asked to switch between colour naming and word reading in a predictable manner in two conditions: cue-absent and cue-present. In the cue-present condition, the stimuli were presented with a circle divided into four equal segments, and, the stimuli were presented in one of the possible segments in clockwise order. The task was cued in a way that participants could work out the required task based on the position of the stimulus. In cue-absent conditions, the circle disappeared, forcing participants to keep track of the task order (to increase the WM load). Wu et al. (2006) failed to find a group difference between cue-absent and cue-present conditions for ADHD participants, suggesting no relationship between task switching and ADHD and that the WM load did not affect those with ADHD any more than control participants.

There may be several reasons for the inconsistent findings. First and most important, the inconsistent results could be the unexplored differences in cognitive performance between ADHD-I and ADHD-C/ADHD-HI and the extent to which the TSP relied on WM. If the TSP has a high WM load component you would expect those with ADHD-I or self-reported inattention to exhibit greater problems with task switching.

Another possible explanation for larger switch costs for ADHD participants when they have been observed, and one that we also explore here in self-reporting adults, and is potentially unrelated to WM capacity, is that the costs were observed under interference load (incongruent stimuli). For example, Cepeda et al. (2000) reported much larger switch costs to incongruent than congruent stimuli for ADHD participants, suggesting that the overall increase in switch costs could be due to the slowed responses on incongruent trials only. The interference view of task switching (Allport et al., 1994; Allport & Wylie, 1999; Wylie & Allport, 2000) suggests that the switch costs (larger RTs to switch than repeat trials) are observed because the persistent activation of the previously activated task-set interferes with the current activation of the new task-set, creating proactive interference. When sufficient time is allowed (long response-stimulus intervals), the activation of the previous task-set decays (*task*-*set inertia*), allowing participants to switch more efficiently due to the minimum amount of interference (Allport et al., 1994).

The interference account also suggests that part of the switch cost derives from interference triggered by the stimulus itself (*task rule congruency*). TSPs may involve unique stimuli for each task (*univalent stimuli*) or both (or more) tasks could be associated with the same stimulus set (*bivalent stimuli*). Smaller switch costs in univalent (unique stimuli for each task) compared to bivalent (two or more tasks associated with the same stimulus set) stimuli have been reported, suggesting that switching is more efficient when the stimulus indicates only one type of task-set (Allport et al., 1994; Rogers & Monsell, 1995; Spector and Biederman, 1976). In sum, it is possible that task-set inertia and/or task rule congruency would play a role in the link between switch costs and ADHD symptoms.

In summary, the literature on ADHD and task switching is inconsistent. We argue that the inconsistent findings could be due to a failure to consider each presentation of ADHD (inattention and hyperactive/impulsive) and/or the type of TSP employed. Given the relationship between WM and inattention, it is likely that inattention will affect task switching performance when there is a WM load. Considering the need for WM to perform preparatory proactive control during task switching, it is reasonable to think that inattentive traits may be related to infrequent engagement of proactive control to prepare for upcoming stimuli due to associated WM limitations (e.g. Elisa et al., 2016; Martinussen et al., 2005). However, where larger switch costs have been reported in those with ADHD, it has been argued that it is a failure to inhibit interference, and not a WM issue, that causes the impaired performance.

## Study 1

In the present study we measured the trait of inattention in undiagnosed adults (along with hyperactivity and impulsivity traits) and its relationship to predictability, interference and WM load during task switching performance. Each participant performed two TSPs: (1) a predictable TSP where participants have to maintain the task order and use this information to prepare in advance for an upcoming repeat or switch trial. In the cue-present condition (low WM load condition), a cue was provided to indicate task order. In the cue-absent condition no additional information was provided and participants had to maintain task order in WM (high WM load condition). The cue-present condition allowed the use of reactive and proactive control, while the cue-absent condition forced participants to rely on proactive control only; (2) an unpredictable TSP where stimuli appeared in an unpredictable manner (forcing the use of reactive control) in long and short RSIs. Long intervals are used to measure the effect of inhibition (task-set inertia) in switch cost.

Given the limitations with WM capacity in inattention at subclinical (Elisa et al., 2016) and clinical levels (Diamond, 2005), one would predict an impairment in maintaining task order and proactively preparing for the next task, resulting in larger switch costs in predictable TSPs, especially when there is no environmental support (the cue-absent condition). In contrast, if inattention was related to a task switching impairment more generally, inattentive traits should predict performance on all TSPs. If instead, an observed switch cost disadvantage was due to a problem with inhibition, there should be an association between one of the core ADHD symptoms in the unpredictable switch cost when the RSI is short. This is because a long RSI confers extra time between the trials to reduce the interference from the previous task-set (Allport et al., 1994).

### Method

#### Participants

Participants aged between 18 and 35 with normal or corrected vision from non-clinical samples were recruited through Bournemouth University’s research participation system and through advertisements. Participants were mainly undergraduate and postgraduate students. Undergraduate students received course credits for their involvement. We collected data from 116 individuals (mean age 20.37, SD 2.87). Initially, sample size was defined by previous research measuring ADHD tendencies on a continuum showing reasonable effect sizes (Elisa et al., 2016).

### Materials

#### Connors’ adult ADHD rating scale: short version (CAARS-S:S)

ADHD tendencies were assessed using CAARS-S:S (Conners, Erhardt, & Sparrow, 1999). The questionnaire requires participants to rate the frequency of the 26 items (symptoms) using a four-point rating scale. Raw scores for inattention, hyperactivity and impulsivity symptoms are transformed into *t* scores to make a comparison across participants. *T* scores range between 28 (lowest) to 90 (highest) calculated based on the age and gender.

#### Predictable task switching paradigm

The task involved the alternative run paradigm (Rogers and Monsell, 1995) and was adapted from Wu et al. (2006). Participants were presented with digits (1, 2, 3, 4, 6, 7, 8 and 9) and asked to decide whether the digit was even/odd (task A) or lower/higher than 5 (task B). The task required participants to press x if the digit was even or lower than five; and press n if the digit was odd or higher than 5. Response mapping was counterbalanced across participants. Digits requiring the same response for both tasks are referred to as being congruent (2, 4, 7, 9). For example, 2 requires the same response (e.g. x) for both tasks since it is even and lower than 5. Stimuli requiring different key responses for each task were referred to as incongruent (6, 8, 1, 3). For example, 6 requires x response for even/odd task while the correct response would be n for lower/higher task. Stimuli were presented in Courier New (bold) 36 points until an appropriate key response or maximum duration of 5000 ms and followed by 150 ms inter stimulus interval.

**Fig. 1:**
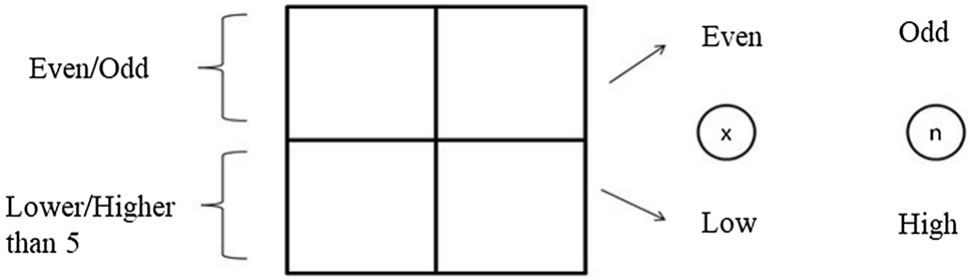
Stimulus cuing frame and instructions for the low load block in Study 1

The task consisted of three blocks: single task block, low load block and high load block. Single task blocks were always presented first to allow participants to establish stimulus–response mappings. In single task blocks, only task A or B was presented consistently within the block. The order of the tasks was randomised across participants. In the low load block, stimuli were presented in a 10 cm by 10 cm square divided into four 5 cm by 5 cm squares (Fig. 1). Stimulus presentation order was always clockwise and as follows: AABB.

The position of the stimulus indicated the task that needed to be performed. The top half of the square indicated the even/odd task while the bottom half indicated lower/higher task. Therefore, task order was always explicitly cued. Participants were also informed about the task order (e.g. even/odd, even/odd, lower/higher, lower/higher) and were told to “switch task every second trial”. The location indicating each task was counterbalanced across participants (e.g. even/odd task was required if the digit was in the top half of the square, and in the bottom half for the other half of the participants). After a response, instead of a blank screen, the square with no stimuli was presented for 150 ms followed by either the reminder screen (for incorrect responses or time-outs) or the next stimulus screen (if the response is correct). There were total of 160 trials with 12 practice trials for the low load block. There were equal numbers of congruent and incongruent trials in each task type (even/odd and lower/higher than 5).

Stimulus–response mapping was counterbalanced across participants with the restriction that even and low; and odd and high was always assigned to the same keys. There were an equal number of participants in each stimulus–response mapping condition for the task type position (top/bottom).

The high load block followed the same instructions with the absence of a square frame. Participants were informed about the task sequence (AABB) and expected to keep track of the sequence. In this block, a reminder provided information about (1) the task instructions (2) the task types for the next two trials. The reason for informing participants about the next two trials was to prevent participants from losing track of the task and to prevent an error leading to a series of errors.

#### Unpredictable task switching paradigm

The stimuli consisted of eight letters (four vowels: A, E, I, U and four consonants: G, M, L, K) and eight digits (four even: 2, 4, 6, 8 and four odd: 3, 5, 7, 9) presented in uppercase 48-point size in Times New Roman (bold). The same two response keys were used for both task sets. That is, participants were to press the c key if the stimulus was a vowel or if it was even; and to press the m key if the letter was a consonant or if the digit was odd. Target responses for vowel/even and consonant/odd stimuli were counterbalanced between participants. A cue was presented for short (50 ms) and long (650 ms) durations immediately before the stimulus (until response or maximum 5000 ms). A reminder of instructions (2000 ms) followed the stimulus screen in the case of incorrect response or time-out.

Single task blocks were always undertaken before the mixed block allowing participants to establish stimulus–response mappings before performing the mixed block. In the single task block participants were always presented with the same type of stimulus (either a letter or a digit). Each task had 64 trials (32 long CSI, 32 short CSI) with 16 practice trials (8 long CSI, 8 short CSI). Only one type of task (A or B) was presented for each block and then the other task was presented.

In the mixed block, digits and letters were presented in a pseudo-random order such that it was not possible to predict the next trial. Mixed blocks consisted of six sequences and reverse versions of each sequence. Therefore, task A and B was counterbalanced within participants. Each sequence involved 17 stimuli. After the first trial there were 8 repetition trials, 4 switch trials and 4 negative priming trials (8 switch trials in total). We controlled the number of negative priming trials because Mayr and Keele (2000) suggested that the reactivation of the recently inhibited task-set is more difficult than if the task set is inhibited a longer time ago. This was because the after effect of inhibition would decay over the time, leading to *negative priming* in the former but not the latter. Using a TSP with three tasks (i.e. A, B, C), they found impaired performance in n-2 repetition (e.g. ABA) compared to n-2 switch (e.g. CBA) trials (see also Arbuthnott and Frank, 2000; Arbuthnott and Woodward, 2002; Arbuthnott, 2005; Hübner, Dreisbach, Haider, & Kluwe, 2003; Koch, Philipp, & Gade, 2006; Schuch and Koch, 2003; Sdoia and Ferlazzo, 2008 for consistent findings). In switch trials, two trials required a switch after two repetitions of the alternative task and two trials occurred after three repetitions of the alternative task. We controlled the number of switch and negative priming trials as they require different levels of inhibition (e.g. Arbuthnott, 2005; Koch et al., 2006; Sdoia and Ferlazzo, 2008). Each sequence was pseudo-randomised with the limitations of: (1) the first trial was always followed by a repeat trial (2) negative priming trials were always presented after a repeat trial or another negative priming trial (3) switch trials were always presented after two or three repeat trials. There were total of 16 sequences (17 trials each) each for short and long RSI conditions. The first two sequences (1 long, 1 short) of the mixed block were practice trials. Before each sequence, participants were informed whether the cue duration would be long or short. After each sequence an information screen was shown indicating that the sequence was completed, and participants had to press space key to proceed, thereby having an interval in between each sequence. Total task duration was approximately 20 min.

### Procedure

The present study included: the CAARS-S:S for measuring traits of ADHD, a predictable TSP and an unpredictable TSP. Tasks were administered in a pseudo-random order with the condition that unpredictable TSP was always presented before the predictable TSP. Since unpredictable TSP required an extra instruction (lower/higher than 5), in order to prevent the confusion, it was always administered first. All versions of unpredictable TSP (S-R mapping) and predictable TSP (even/odd first, low/high first; and S-R mapping) were counterbalanced across participants.

### Results

#### Sample

**Fig. 2:**
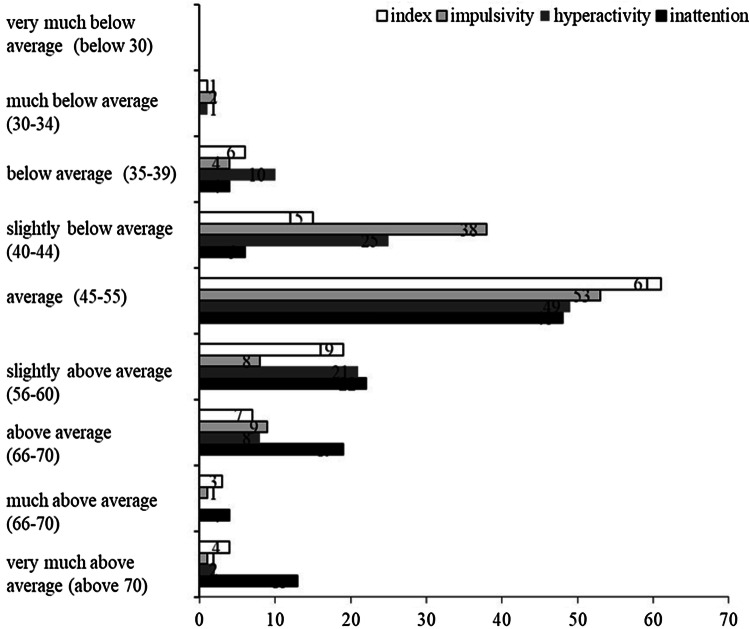
Number of participants in Study 1 falling into each category and the corresponding *T* scores in brackets based on CAARS-S:S guidelines

Scores from CAARS revealed that 23 participants scored above average on the ADHD index (*M* = 51.24, SD 8.46). For individual symptoms, the number of participants that scored above the average was 35 for inattention (*M* = 55.78, SD 9.34), 26 for hyperactivity (*M* = 49.65, SD 8.21) and 13 for impulsivity (*M *= 48.02, SD 7.86). The number of participants scoring in each category provided by the CAARS-S:S guidelines are reported in Fig. 2. Raw scores are transformed into standardised T-scores so that all sub-scales have mean of 50 and standard deviation of 10. T scores range between 28 (lowest) and 90 (highest) calculated based on the age and gender. Our mean and standard deviations for impulsivity, hyperactivity and index scores were within half a standard deviation from the proposed mean and standard deviations for CAARS-S:S. The mean for the inattention scores was half a standard deviation above the proposed mean but still within the confidence interval values. One participant also reported previous ADHD diagnosis, whereas two participants preferred not to state. Please see Fig. 2 for detailed participant characteristics.

##### Analysis of general switch costs

RTs for incorrect responses and trials following incorrect responses and the data points two standard deviation above and below the mean (1.4%) were removed before the analysis.

**Fig. 3:**
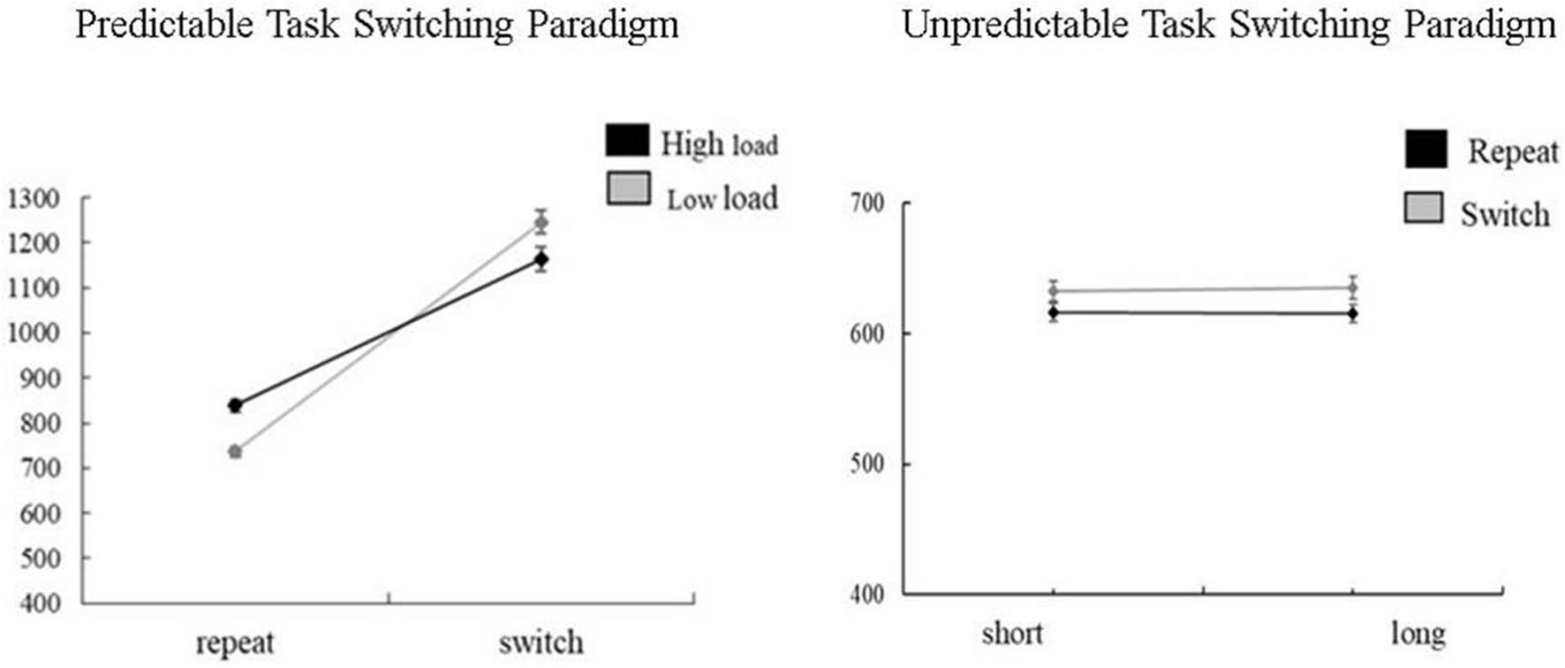
Average RTs in Study 1 based on the conditions of the stimuli in predictable and unpredictable task switching paradigms

*Predictable task switching paradigm*. We conducted a 2 (condition: low load, high load) × 2 (transition: repeat, switch) repeated measures ANOVA to evaluate the switch cost (see also Fig. 3). A transition main effect indicated that overall RTs were higher on switch (*M* = 1189.76, SE 22.05) than repeat (*M* = 785.31, SE 9.94) trials [*F*(1, 102) = 457.12, *p* < 0.001, *η*^2^ = 0.82] while the condition main effect was not significant, *F*(1, 102) = 2.02, *p* = 0.158, *η*^2^ = 0.02.

There was a significant condition × transition interaction, *F*(1, 102) = 44.34, *p* < 0.001, *η*^2^ = 0.30. Bonferroni corrected paired samples *t* tests revealed that in the low load condition, responses to switch trials (*M* = 1226.99, SE 24.93) were slower than repeat trials (*M *= 738.74, SE 10.46), *t*(108) = 23.23, *p *< 0.001. Same effect was observed in the high load condition [switch: *M* = 1156.29, SE 27.51; repeat: *M* = 839.26, SE 14.46), *t*(106) = 13.91, *p* < 0.001].We also found that in the high load condition, repeat trials were slower [*t*(104) = 6.87, *p *< 0.001] while switch trials were faster compared to the low load condition [*t*(109) = 2.41, *p *= 0.017]. However, this difference did not reach significance following Bonferroni correction (*P*_corrected_ = 0.013). To test the effect of WM load on task switching, we compared the switch cost across conditions. Paired samples *t* tests revealed that switch costs were higher in the low load (*M* = 474.82, SE 20.54) compared to the high load (*M* = 322.98, SE 22.69) condition *t*(101) = − 6.54, *p *< 0.001.

We next analysed the switch and repeat RTs for congruent and incongruent conditions separately. Bonferroni corrected paired samples *t* tests revealed that RTs to switch trials were longer than repeat trials in all conditions: for low load, switch trials took longer than repeat trials for congruent [switch: *M *= 1192.78, SE 25.41; repeat: *M *= 733.81, SE 11.29, *t*(110) = 21.64, *p *< 0.001] and incongruent stimuli [switch: *M *= 1268.67, SE 25.38; repeat: *M *= 751.95, SE 12.19, *t*(107) = 23.60, *p *< 0.001]. Similarly, for high load, switch trials took longer than repeat trials for congruent [switch: *M* = 1116.60, SE 28.78; repeat: *M *=844.72 = 839.26, SE 16.83, *t*(109) = 11.17, *p *< 0.001] and incongruent stimuli [switch: *M* = 1221.20, SE 29.18; repeat: *M* = 834.28, SE 15.33, *t*(107) = 15.06, *p *< 0.001].

*Unpredictable task switching paradigm*. We conducted a 2 (RSI: short, long) × 2 (transition: repeat, switch) repeated measures ANOVA to evaluate the switching cost and the effect of RSI. A transition main effect indicated that overall RTs were higher in switch (*M* = 627.12, SE 6.27) than repeat (*M* = 602.37, SE 6.27) condition, *F*(1, 89) = 80.61, *p* < 0.001, *η*^2^ = 0.48. However, the RSI main effect [*F*(1, 89) = 0.41, *p* = 0.525, *η*^2^ = 0.01] and RSI × transition interaction [*F*(1, 89) = 2.69, *p* = 0.104, *η*^2^ = 0.03] were not significant, indicating that the RSI manipulation was not effective.

#### Inattention and task switching

We examined the bivariate correlations between the independent and dependent variables (Table 1). Pearson correlation coefficients revealed significant positive correlations between inattention scores and switch cost when the tasks were predictable. Inattention was correlated to predictable switch costs in low load (*r* = 0.19, *p* =0.05) and high load (*r* = 0.26, *p *< 0.01) conditions. We also measured the switch costs separately for stimulus congruency: in the low WM condition, inattention significantly correlated with the switch costs when the stimuli were incongruent (*r* = 0.22, *p *= 0.03) but this correlation was no longer significant in the high WM load condition. In contrast, in the high WM condition, inattention was correlated with the switch costs of congruent (*r* = 0.19, *p *= 0.05) but not incongruent stimuli. Furthermore, inattention was not significantly correlated with the switch costs when the task order was unpredictable (short RSI: *r* = 0.02, *p *= 0.88; long RSI: *r* = 0.08, *p *= 0.41).

**Table 1:** Correlations between variables in Study 1

	Mean	SD	1	2	3	4	5	6	7	8	9	10	11	12
1. Inattention	55.78	9.34	–											
2. Hyperactivity	49.65	8.21	0.39**	–										
3. Impulsivity	48.02	7.86	0.45**	0.45**	–									
4. Index	51.24	8.46	0.71**	0.66**	0.72**	–								
5. Unpredictable switch cost (short RSI)	19.77	28.91	0.02	0.03	− 0.06	− 0.05	–							
6. Unpredictable switch cost (long RSI) (log transformed)	1.43	0.44	− 0.01	0.14	0.11	0.11	0.23*	–						
7. Predictable switch cost (low load)	482.27	211.39	0.19*	0.04	0.13	0.08	− 0.01	0.14	–					
8. Predictable switch cost (high load)	317.03	235.74	0.26**	− 0.03	0.08	0.11	0.43**	0.14	0.24*	–				
9. Congruent stimuli switch cost (low load; predictable)	458.97	223.45	0.15	− 0.05	0.09	0.06	0.12	0.16	0.95**	0.42**	–			
10. Incongruent stimuli switch cost (low load; predictable)	516.72	227.50	0.22*	0.10	0.19	0.12	0.08	0.12	0.93**	0.43**	0.79**	–		
11. Congruent stimuli switch cost (high load; predictable)	271.89	255.22	0.19*	0.02	0.07	0.13	0.10	− 0.09	0.31**	0.87**	0.27**	0.31**	–	
12. Incongruent stimuli switch cost (high load; predictable)	286.92	266.92	0.15	− 0.07	0.03	0.06	0.12	0.05	0.42**	0.81**	0.39**	0.42**	0.57**	–

*p < 0.01, **p < 0.05

We ran multiple regression analysis to investigate the role of ADHD traits when explaining switch costs. We also used Bayes Factors (B) to assess the strength of evidence in support of hypotheses when the p value for the predictors was not significant. We followed Dienes (2014) to assess the strength of evidence in support of hypotheses when the p value for the predictors was not significant. Where a Bayes Factor is given, we modelled the predictions of the theory of some evidence for a relationship with a half-normal whose mean and standard deviation values were taken from Cepeda et al. (2000): experiment 1 for predictable and experiment 2 for unpredictable TSPs due to the similarity of the procedure to our study. We used the value of r square (coefficient of determination) to calculate Bayes Factor where the regression model was non-significant (using BayesFactor package of R software, Liang, Paulo, Molina, Clyde, & Berger, 2008).

For predictable switch costs under high load, the multiple regression analysis revealed that the model (Table 2) explained 9% of the variation, *F*(3, 106) = 3.27, *p *= 0.02. Hyperactivity (*p* = 0.131, *B*_H(0, 0.422)_ = 0.04) and impulsivity (*p* = 0.926, *B*_H(0, 0.422)_ = 0.04) were non-significant predictors with Bayes Factors providing strong evidence for the null. Thus, inattention was the only predictor of the predictable switch cost under high WM load. The regression model where ADHD traits predict the switch costs in the low load condition of the predictable task was not significant and the Bayes Factor provided strong evidence for the null *F*(3, 107) = 1.57, *p *= 0.20, *B* = 0.16.

**Table 2:** Summary of regression model for inattention, hyperactivity and impulsivity scores on switch cost in high working memory load condition in Study 1

Variable	*b*	SE*b*	*β*	*T*	*R*^2^	Adjusted *R*^2^	Semi-partial correlation
Inattention	7.75	2.68	0.32	2.89**	0.09	0.06	0.27
Hyperactivity	− 4.74	3.12	− 0.17	− 1.52			− 0.15
Impulsivity	0.31	3.33	0.01	0.09			0.01

*p < 0.05, **p < 0.01

Due to the significant correlations, we ran multiple linear regression models to investigate whether inattention, hyperactivity and impulsivity predicted the switch cost based on congruency. Switch cost to incongruent stimuli in the low load condition [*F*(3, 107) = 2.13, *p *= 0.101, *B* = 0.31], and, the switch cost to congruent stimuli in the high load condition [*F*(3, 109) = 1.47, *p *= 0.227, *B* = 0.04] yielded non-significant results. Bayes values also revealed strong evidence for the null hypothesis of no difference.

### Discussion

We employed predictable and unpredictable task switching paradigms (TSPs) to investigate whether self-reported inattention is related to a general task switching impairment, a limitation in inhibition, or, in line with research showing a relationship between inattention and working memory (WM), an impairment specifically related to predictable task switching. The predictable TSP required participants to keep the task order available in WM and use this information to predict the next task to be performed, a form of proactive control. In the unpredictable TSP, the task order changed in a pseudo-random order, not allowing participants to prepare or to use previous information to work out the upcoming task. Therefore, the unpredictable task primarily required reactive control.

Given the negative relationship between the WM capacity and inattention even in subclinical populations (Elisa et al., 2016; Lui and Tannock, 2007), we predicted that inattentive traits would be more related to the higher switch costs during predictable switching as the task requires the use of proactive control. Moreover, it was predicted that the relationship between inattentive traits and switch costs would be stronger when the task involved a higher WM load (in the cue-absent condition). As predicted, we found that inattentive traits predicted greater switch costs in the predictable TSP but only under high WM load conditions. Bayes values provided evidence towards no relationship between hyperactivity/impulsivity and the switch cost under predictable task switching conditions and for no relationship between the ADHD-related traits and switch costs in the unpredictable TSP.

It is interesting that inattention was related to poorer performance on what is essentially an easier task since the task changed in a predictable manner and thus it was possible to prepare the correct task set in advance. Such impairment fits well with the problems with planning and organisation associated with inattention. To benefit from the preparation, participants had to keep the task order available in WM and use this information to identify the next task. Our results suggest that those with inattention are specifically impaired at this preparatory activity. Whilst we found that inattentive traits were positively correlated with switch costs in the cue-present condition, this relationship was not predictive.

We also measured switch costs separate for congruent and incongruent stimuli since the literature suggested that larger switch costs for ADHD may be driven by the switch costs for incongruent (RTs to incongruent switch trials—RTs to incongruent repeat trials) rather than congruent (RTs to congruent switch—RTs to congruent repeat trials) stimuli (Cepeda et al., 2000). As noted above the ADHD index score did not predict any switch costs in our study contrasting with the findings from Cepeda et al. (albeit in a subclinical population). However, our analysis revealed that inattention was correlated to incongruent trial switch costs, but only under low WM load. Inattention was also correlated with congruent trial switch costs, but only under *high* WM load. However, inattention did not predict the magnitude of either of these indicating that in our data at least inattention does not lead to increased switch costs as a result of trial congruency.

Consistent with Liefooghe et al. (2008), switch costs were modified by WM. In the predictable TSP, responses to the repeat trials were longer in the high compared to the low WM load condition, indicating an effect of WM load in the expected direction. However, responses to the switch trials were quicker in the high (cue absent) compared to the low (cue present) WM load condition. We also found that the switch costs decreased in the high compared to low WM load condition (Fig. 3). This could be due to the type of control executed by the participants. The cue-present condition involved proactive and/or reactive control depending on the strategy (keeping track of the order or benefiting the cue) to perform the task; keeping track of the task order allowed participants to prepare in advance as they figure out the next stimuli from the maintained task order and this could happen before the next stimulus appears. Utilising the cue did not allow advanced preparation. That is, in the cue-present condition, participants could choose from the two strategies for responding. The cue-absent (high WM load) condition, however, forced participants to keep track of the order which may have encouraged advance preparation, thereby reducing response times to the switch trials in the cue-absent (high WM load) compared to the cue-present (low WM load) condition. This supports the notion that inattentive traits uniquely predicted the switch costs when WM was needed to perform proactive control for advanced preparation (cue-absent condition of predictable TSP).

To summarise the results from Study 1, we found that only inattentive traits significantly predicted task switching performance. This was only observed when switching was predictable and trial order was not indicated by a cue, suggesting that it was the requirement to track task order and utilise proactive control that led to larger switch costs in those with high levels of inattention. Furthermore, none of the ADHD-related tendencies were correlated to switch costs in an unpredictable TSP. These findings indicate that the impairment in WM associated with inattention can lead to task switching impairments and that the failure to observe a consistent relationship between ADHD and task switching performance in previous studies is likely due to the failure to consider the differential influence of the core symptoms of inattention, hyperactivity and impulsivity. However, these conclusions are mitigated by certain limitations in the experimental design. First, the ability to keep track of the task order and use proactive control was confounded in the present study. Second, the predictable and unpredictable paradigms differed in several ways: (1) the stimuli in the predictable TSP were bivalent while the unpredictable TSP had univalent stimuli; (2) the RSI was manipulated in the unpredictable TSP only leading to differences in time constraints between the predictable and unpredictable tasks. Finally, whilst there are a number of participants falling into each category that is spread normally (see Fig. 2), the hyperactive and impulsive scores were numerically more restricted than the inattention scores, which could have reduced the likelihood of observing a relationship between these symptoms and task switching performance (although assuming a linear relationship, we believe this would not have had a significant impact on the results).

## Study 2

Study 1 revealed that self-reported inattentive traits uniquely predicted higher switch costs in a predictable task switching paradigm in which working memory (WM) was needed to track task order while unpredictable switching was not related to any of the ADHD symptoms. However, the tasks differed more than in predictability. Therefore, in the present study we addressed the methodological issues raised above using bivalent stimuli in both the predictable and unpredictable TSP and eschewing an RSI manipulation. In addition, the present study also sought to identify factors that might lead to a relationship between inattention and predictable task switching performance.

For those participants high in inattention the factor limiting performance in Study 1 was either the need to keep track of the order of repeat and switch trials or the need to utilise preparatory proactive control when the order was known. Given the predictable and basic nature of the sequence, the participants should have been able to take advantage of the simple sequence to improve their performance and proactively prepare for each upcoming trial. Inattention did not predict performance when there was environmental support for tracking task order. Clearly when the location cued the task was to be performed, the need for a contribution from working memory to track task order was reduced. Such a result could be explained by either an impairment in working memory or in the use of proactive control. In Study 1 these factors were confounded.

In the present experiment, participants were asked to complete five blocks of task switching where task order was either predictable (a trackable sequence engaging WM) or it was unpredictable. The task was cued with two frames (an advanced cue presented before the stimulus and stimulus cue presented with the stimulus) in black, red or blue. The coloured cue indicated which task to perform while black cue was uninformative. Moreover, in some blocks coloured advanced cue enabled participants to engage in proactive control and some blocks included a coloured stimulus cue that permitted participants to utilise reactive control to select the correct task set. The five block types were the following: (1) a predictable task switching order with black advanced cue on any trial but a coloured stimulus cue indicating the task to be performed; this condition is referred to as Order PC/RC because the predictable order permitted the use of proactive control and the stimulus colour cue permitted the use of reactive control; (2) a random task switching order with coloured advanced cue on every trial and a coloured stimulus cue indicating the task to be performed; this condition is referred to as Random PC/RC because the order was random and the advanced cue permitted the use of proactive control and the stimulus colour cue permitted the use of reactive control; (3) a predictable task switching order with black advanced cue and black stimulus cue indicating the task to be performed; this condition is referred to as Order PC because the predictable order permitted the use of proactive control (this condition is the condition most similar to the high WM load condition of Study 1); (4) an unpredictable task switching order with a coloured advanced cue on every trial but black stimulus cue indicating the task to be performed; this condition is referred to as Random PC because the order was random and the advanced cue permitted the use of proactive control; (5) an unpredictable task switching order in which a coloured stimulus cue permitted the use of reactive control; the condition is referred to as Random RC. This design permits the de-confounding of working memory load and proactive control. If inattention was related to impairment in the use of proactive control, it would be related to performance in any block that presents an advanced cue (Random PC/RC or Random PC). If inattention was related to working memory impairments, it would be related to performance in any block/condition that has a predictable sequence and involves the need to keep track of the order of switch and repeat trials (Order PC/RC or Order PC), but especially Order PC where no other cue is provided about which task to perform (thereby replicating Study 1). If inattention was related to an impairment in reactive control it would affect performance most clearly in the random RC block.

The de-confounding of working memory and proactive control is a necessary step in identifying the determining factor producing the relationship between predictable task switching and inattention. However, in the present study we also considered another potential contributor to this result. Specifically, Elisa et al. (2016) reported a relationship between working memory performance and subclinical symptoms of ADHD. The only working memory related task that was uniquely related to inattention was a letter monitoring task that measured the tendency for goal neglect. In goal neglect, although instructions are understood and not forgotten (a representation of the task is created; Duncan, Emslie, Williams, Johnson, & Freer, 1996; Duncan et al., 2008) participants behaviourally fail to follow these instructions (Duncan et al., 1996). Duncan et al. explained the occurrence of goal neglect with reference to competition in the task model: in order to perform complex tasks, individuals need a body of knowledge composed of all relevant facts and instructions (*the task model*) where the cue-action mappings with sufficient saliency are constructed. The model should be organised into small chunks of information to be retrieved when relevant triggering conditions occur. As the information in the task model is increased (e.g. by increasing the complexity in the task instructions), multiple task components compete to be represented. Due to limited capacity in some individuals, some of the task components are too weakly represented to be used when needed, resulting in goal neglect (Duncan et al., 1996, 2008). When asked, participants can re-report the instructions in full, but it is the use of components of the task model during task performance that is impaired. Goal neglect has been linked to the lapses in WM (Kane and Engle, 2003; Duncan, Schramm, Thompson, & Dumontheil, 2012) and fluid intelligence which is related to cognitive control functions (Duncan et al., 1995; Kane and Engle, 2003; Marshalek, Lohman, & Snow, 1983; Oberauer, Süß, Wilhelm, & Wittman, 2003). Along with various measures of WM, Elisa et al. (2016) also measured the link between inattention, hyperactivity and impulsivity and goal neglect based on the notion that those with inattention have problems receiving verbal instructions. They showed that inattention was uniquely related to goal neglect even when controlling for fluid IQ.

The original conception of goal neglect has been influential and other researchers have extended the concept. De Jong (2001) proposed the notion of the *failure to engage hypothesis* and referred to this as goal neglect. De Jong (2000) argued that the residual switch cost, a cost, even after being given time to prepare for an upcoming trial, remains because participants occasionally fail to engage and maintain goal-related preparation (but see Mayr and Keele, 2000; Rogers and Monsell, 1995; Rubinstein, Meyer, & Evans, 2001 for an alternative view). According to the failure to engage hypothesis, individuals sometimes fail to take the opportunity to perform preparation. Given the link between inattention and increased reports of goal neglect, we hypothesised that the link between inattentive traits and increased predictable switch costs could be moderated by goal neglect. If this is supported by the data, then it would support the notion that goal neglect is an important contributor to the experience of inattention.

### Method

#### Participants

As with Study 1 we collected data from 120 (different) individuals (*M* = 20.55, SD 2.31). Participants aged between 18 and 33 with normal or corrected vision from non-clinical samples were recruited through Bournemouth University’s research participation system and through advertisements. Participants were mainly undergraduate and postgraduate students. Undergraduate students received course credits for their involvement.

#### Materials

##### Adult ADHD self report scale (ASRS)

We used ASRS to measure ADHD-related traits (Adler, Spencer, Faraone, Kessler, Howes, Biederman, & Secnik, 2006; Kessler et al., 2005). In order to show that the results of the first study were not specific to the scale employed (CAARS-S:S), in the current study, ASRS was used as an alternative scale. ASRS includes total of 18 items consisting the ADHD symptoms of Diagnostic and Statistical Manual of Mental Disorders Fourth edition (DSM-IV). There are nine items indicating inattentive symptoms (1, 2, 3, 4, 7, 8, 9, 10, 11) and nine items indicating hyperactive/impulsive symptoms (5, 6, 12, 13, 14, 15, 16, 17, 18). ASRS asks participants how often a particular symptom of ADHD has occurred to them over the past 6 months on a five-point response scale ranging from “never” (0), “rarely” (1), “sometimes” (2), “often” (3), to “very often” (4). The ASRS was scored by averaging the participants’ ratings across the responses in each symptom cluster, providing us a continuous scale (Overbey et al., 2011; Whalen, Jamner, Henker, Gehricke, & King, 2003).

##### Task switching paradigm

**Fig. 4:**
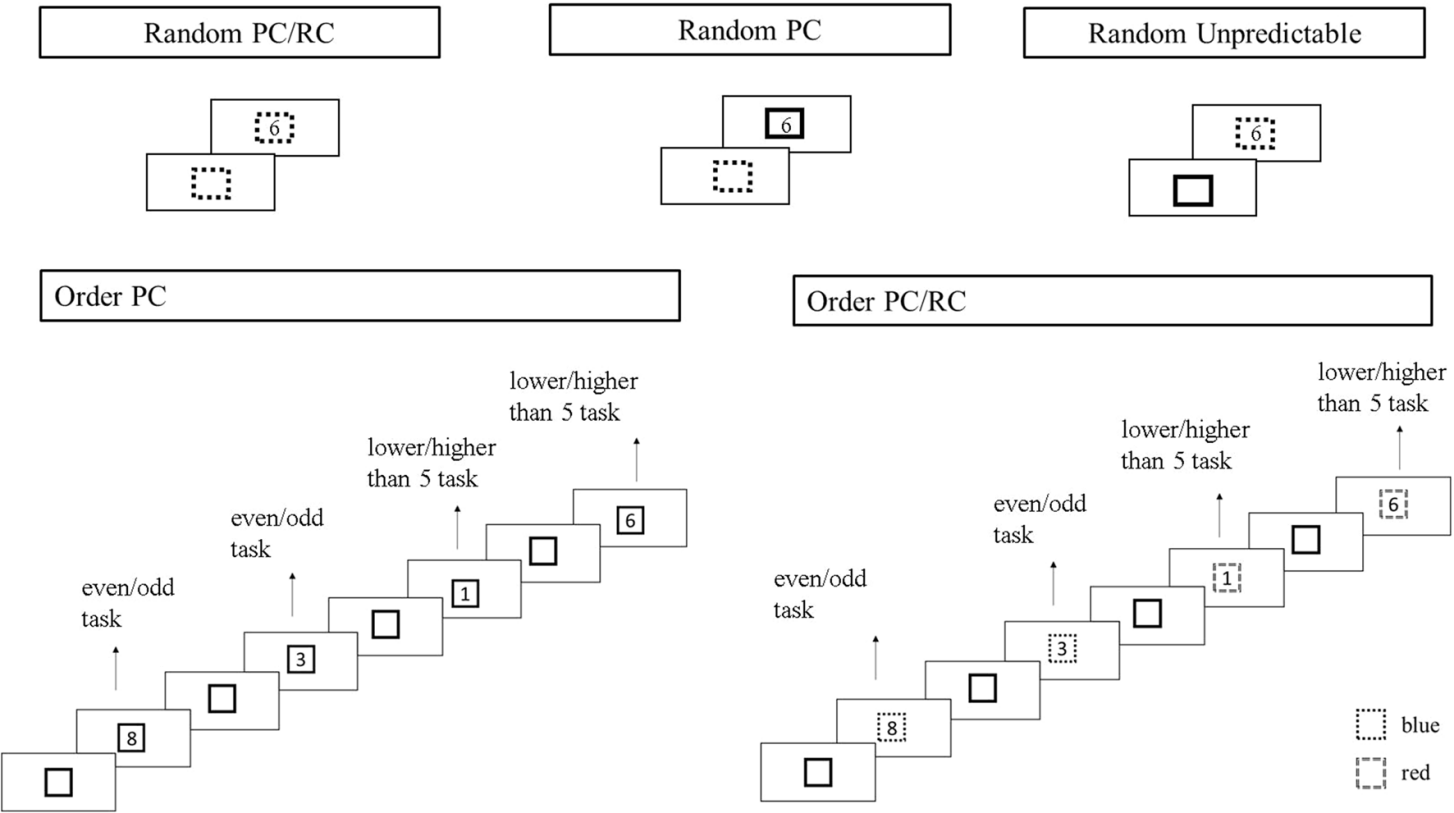
Example demonstration for the blocks of mixed condition in the task switching paradigm in Study 2

The task required participants to perform two types of tasks: participants were required to decide if the digit was even or odd (even/odd task) or if the digit was lower or higher than five. Available responses (‘z’ and ‘m’) counterbalanced across participants. The task comprises two conditions where the pure condition required only one type of task throughout the block while the mixed condition required frequent switches between two tasks. There were two blocks for the pure condition (one block for each type of task) presented in random order. Pure blocks were designed to make participants familiar with each type of task and learn stimulus–response associations. It was also aimed to test participants’ ability to perform each task. Participants performed total of 16 practice and 64 experimental trials for the pure condition. The Mixed Condition included five blocks presented in random order. Each block included 16 practice and 96 experimental trials. Stimuli consisted of digits between one and nine except five, presented in Courier New Bold in 36 points (bold). Before the stimulus presentation, a square with 2.8 cm length appeared as a cue (advanced cue) and stayed on the screen as a frame for the stimulus (stimulus cue). Depending on the block, the frame was either red, blue or black. The colours red and blue indicated the task to be performed (counterbalanced across participants). At the beginning of each block, participants were asked to make a key press when they were ready. A 2000-ms blank screen followed the key press. Each trial started with the square frame; then, the stimulus was presented inside the frame after 250 ms. The stimulus was present until the response (maximum response duration was 5000 ms). Following an error, a reminder for the rules appeared on the screen for 1200 ms. Please see Fig. 4 for a depiction of the sequence of events.

In the *Random PC/RC block,* both the advanced cue and the stimulus cue were coloured indicating the task to be performed. Hence, participants had the opportunity to attend the advanced cue or the stimulus cue. The advanced cue allowed advanced preparation and the use of proactive control, while the stimulus cue did not. Instead, the stimulus cue required participants to engage in reactive control where the cognitive processes are triggered by the stimulus presentation. The tasks were presented in a random order.

In the *Order PC/RC block* followed the same procedure as the previous blocks, except that the tasks were presented in a set order (AABB…). The stimulus cue was presented in red or blue, indicating the task to be performed. Therefore, participants could either keep track of the task order or wait for the stimulus cue to figure out the required task. Keeping track allowed advanced preparation (proactive control) while attending the stimulus cue required reactive control.

In the *Random PC block,* the advanced cue was presented in red or blue, indicating the upcoming task. The frame then turned to black. Since participants had to focus on the advanced cue to figure out the next task, they were strongly encouraged to engage in advanced preparation. The tasks were presented in a random order.

The *Order PC block,* similar to Order PC/RC block, the tasks were presented in a set order (AABB…). Both the advanced cue and the stimulus cue were always black, forcing participants to keep track of the task order to find out the task to be performed. Keeping track of the task order allowed participants to know the upcoming task before stimulus presentation, hence strongly encouraging advanced preparation.

Finally, in the *random RC* condition, tasks were in random order and were indicated by the stimulus cue, allowing only the use of reactive control. Therefore, this block involved unpredictable switching.

##### Automated operation span task

Automated version of operation span task taken from Unsworth et al. (2005) was used as a measure of working memory capacity. The task required participants to remember the letters and solve mathematical problems in between as the distraction (Unsworth et al., 2005). Mathematical problems and letters were presented one at a time in the centre of the screen. Participants reported the sequence of letters by choosing among possible letters from a 4 by 3 matrix of possible letters (F, H, J, K, L, N, P, Q, R, S, T, and Y). For the mathematical operations, participants were told to solve the mathematical operation as quickly as possible and press a mouse button when ready. Next, participants were asked to report if the number presented on the screen is the correct solution of the mathematical problem by clicking on either the true or the false button.

Participants completed a practice session with simple letter span following another block of 15 mathematical problems only. In the experimental condition, letters appeared on the screen for 800 ms while recall phase was untimed. After the recall, an accuracy feedback for both operations was provided. Following the practice sessions for letter recall and mathematical problems, participants had a final practice combining both operations, identical to the experimental condition. In the experimental condition, sequences of mathematical problems and letters were presented. There were three sets from each possible set size (3–7 letters to remember and mathematical problems to solve). Thus, in total, 75 letters and 75 mathematical problems were presented. Scores are calculated by adding the number of letters recalled in the correct order (also known as the partial score; Turner & Engle, 1989). Participants below the 85% accuracy were not included in the analysis to ensure that participants were attempting to perform both operations.

##### Letter monitoring task

**Fig. 5:**
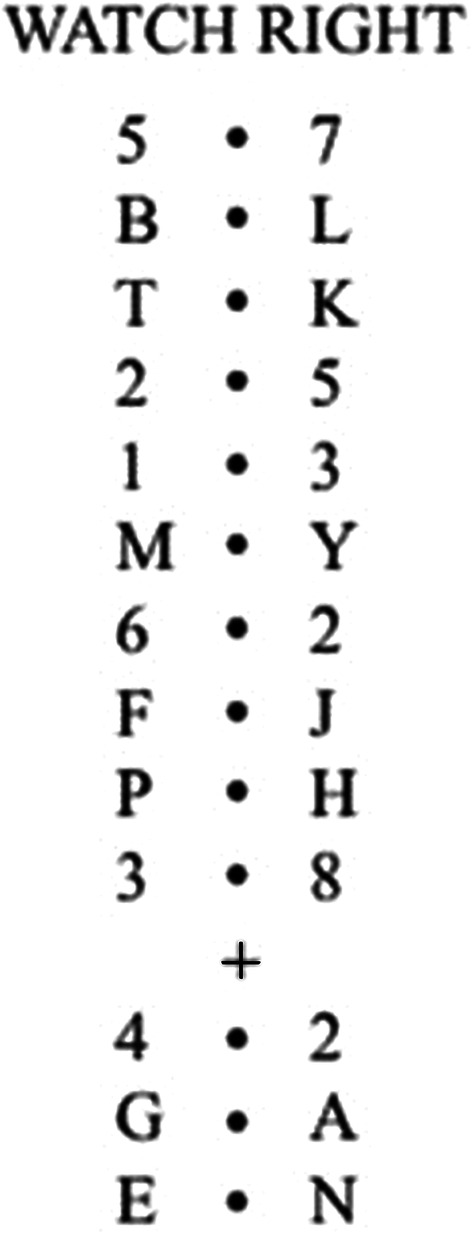
An example demonstration of a letter monitoring task trial (used in Study 2) taken from Duncan et al. (1996). Starting from the top to bottom, “Watch RIGHT” message (1 s) is followed by the pairs. Each pair is presented in a separate screen for 200 ms with a blank interval of 200 ms

The letter monitoring task was taken from Duncan et al. (1996) as a measure of goal neglect. In the letter monitoring task, participants are presented with pairs of letters or digits on the left and right side of a central dot. The task is to ignore the digit trials and read aloud the letters on the directed side on the letter trials. Each trial included the presentation of 13 pairs of digits/letters (Fig. 5). Digits were chosen from the set 1–8, and letters were randomly chosen without replacement from the letters of the alphabet (except D, I, O, V, and W). Following the instructions of Duncan et al., participants were first prompted by a “READY?” message. Following the participant’s positive response via verbal report, the experimenter made a key press to initiate a 500-ms blank interval after which the practice trial began. Each trial started with the presentation of the instruction “WATCH LEFT” or “WATCH RIGHT” for 1 s indicating the side from which the participant was required to report the letters. The message was followed a by a further 1-s interval for the participant to get ready for the upcoming stimulus sequence. Each stimulus screen consisted of either a pair of digits or letters presented for 200 ms followed by a blank interval of 200 ms. Initially, there were ten pairs. After the 10th pair, the cue with a “+” or “−” symbol was presented in the centre of the screen for 200 ms. A “+” sign indicated to the participant that they must attend to the right while “−” sign indicated to attend to the left side of the dot (again reporting only from trials with letters). Following a further 200 ms, three more pairs were presented. After the symbol, the first pair was always digits and the last two were always letters. Thus, each trial had total of 13 pairs of digits or letters. Please see Fig. 5 for an example trial. A scoring sheet with correct answers was prepared for the experimenter in advance to manually record the participant’s responses.

To ensure that the cue was remembered correctly, pieces of paper were placed on the appropriate side of the computer monitor with “PLUS” (for the right) and “MINUS” (for the left) signs written on them. All participants were instructed to: (1) read aloud the letters and ignore the digits; (2) initially report from the side instructed by the message on the screen (until the cue is presented); (3) then use the cue (+ or − sign) to attend the correct side for the next three pairs. The task comprised three blocks of 12 experimental trials (with 13 pairs presented in each trial) with 3 sub-blocks (4 trials each) within each block. Participants also received a practice trial which was repeated until at least one letter was reported from either (correct or incorrect) side and the “+/−” cue was reported accurately. For some trials, participants had to change the attended side (e.g. a WATCHLEFT message followed by the + cue which indicates attending right) while others did not require a change (e.g. a WATCHLEFT message followed by the − cue which indicates attending left). To equalise the number of trials with change and no change, in each successive trial of four, there was one “WATCH LEFT” followed by a “−” cue, one “WATCH LEFT” followed by a “+” cue, one “WATCH RIGHT” followed by a “−” cue, and, one “WATCH RIGHT” followed by a “+” cue presented in random order. Participants were asked to repeat the rule again between each block. Instructions for the task were provided following Duncan et al.’s (1996) instructions.

*Scoring* A correct response was defined as the following of the cue instruction. Participants received a score of 1 for each letter reported from the correct side. A perfect trial included a score of two with two letters reported from the correct side. There were two requirements for scores to be counted. First, for the trials to be valid, participants had to report at least three letters from the appropriate side indicated by the initial message (“WATCH LEFT”/“WATCH RIGHT”). This was to ensure that participants attended to the side indicated by the initial message. Second, participants had to report at least one valid change and one valid no-change trial to pass each sub-block. The final score was computed by the sum of the each passed sub-block. Scores indicate to what extend a participant’s performance was affected by the cue.

##### Raven’s standard progressive matrices (SPM; shortened version)

We used Raven’s standard progressive matrices to measure fluid intelligence as it is related to goal neglect and working memory performance (Duncan et al., 2008; Kane, Hambrick, & Conway, 2005). Participants SPM scores were also entered into the regression analysis as a predictor variable to ensure the predictive value of goal neglect and working memory were not simply as result of the variance these measures share with fluid intelligence. In the shortened Raven SPM (Bouma, Mulder, & Lindeboom, 1996), three sets of items from the original version (Sets B, C, and D) were administered as an indicator of fluid intelligence. Each item consisted of a matrix of black and white elements composing an overall pattern (rule). Participants were asked to complete this pattern by choosing the correct missing element among multiple possible options. There was no time limit in completing the test. Each item was scored either 1 (correct) or 0 (incorrect). Administration and scoring were carried out based on the guidelines provided in the SPM manual (Raven, 1938).

### Procedure

The present study included: the ASRS for measuring ADHD related tendencies, Task Switching Paradigm, OSPAN as a working memory measure, Letter Monitoring Task as a measure of goal neglect (and Feature Matching Task), and SPM as a control measure of fluid abilities for the goal neglect measures. After providing the participant information sheet and the informed consent, tasks were administered in counterbalanced order.

### Results

Scores from ASRS ranged between zero and four. Mean scores and standard deviations for ASRS are reported in Table 3. The mean scores of inattention were again higher than those for hyperactivity/impulsivity and total ADHD scores but this difference was within 1 standard deviation above and below the mean. Outliers 2SD above and below the mean were removed for SPM (3.33%), Random PC/RC (5%), Order PC/RC (5%), Random PC (5.8%), Order PC (5.8%), and Random RC (5%) due to non-normal distributions. The OSPAN scores for the participants scoring below 85% accuracy on math questions were also removed (7.5%). Participants were only excluded for specific parts of the experiment if their performance was deemed as outliers for that part of the experiment. Total of 52 out of 1200 data points (4.33%) were removed across all analyses.

**Table 3:** Correlations between variables in Study 2

	*N*	*M*	SD	1	2	3	4	5	6	7	8	9	10	11
1. Inattention	120	2.06	0.69	–										
2. Hyp/Imp	120	1.45	0.73	0.51**	–									
3. ADHD total	120	1.76	0.62	0.86**	0.88**	–								
4. SPM	116	31.27	2.67	− 0.02	− 0.02	− 0.02	–							
5. Letter monitoring	120	4.48	4.27	− 0.19*	− 0.02	− 0.11	0.31**	–						
6. OSPAN	111	55.45	11.70	− 0.29**	− 0.22*	− 0.29**	0.07	0.29**	–					
7. Random PC/RC	114	434.80	246.58	0.20*	− 0.01	− 0.01	− 0.02	− 0.26**	− 0.13	–				
8. Order PC/RC	114	429.51	219.33	0.22*	0.09	0.18	0.15	− 0.22*	− 0.11	0.37**	–			
9. Random PC	113	205.44	223.09	− 0.03	− 0.08	− 0.07	− 0.06	0.10	0.06	0.31**	0.21*	–		
10. Order PC	113	241.81	236.81	0.05	0.04	0.05	0.40**	0.07	− 0.02	0.05	0.31**	0.04	–	
11. Random	114	352.69	207.48	0.09	− 0.02	0.04	0.20*	− 0.17	− 0.10	0.44**	0.53**	0.21*	0.17	–

*p < 0.01, **p < 0.005

#### Analysis of switch costs

**Fig. 6:**
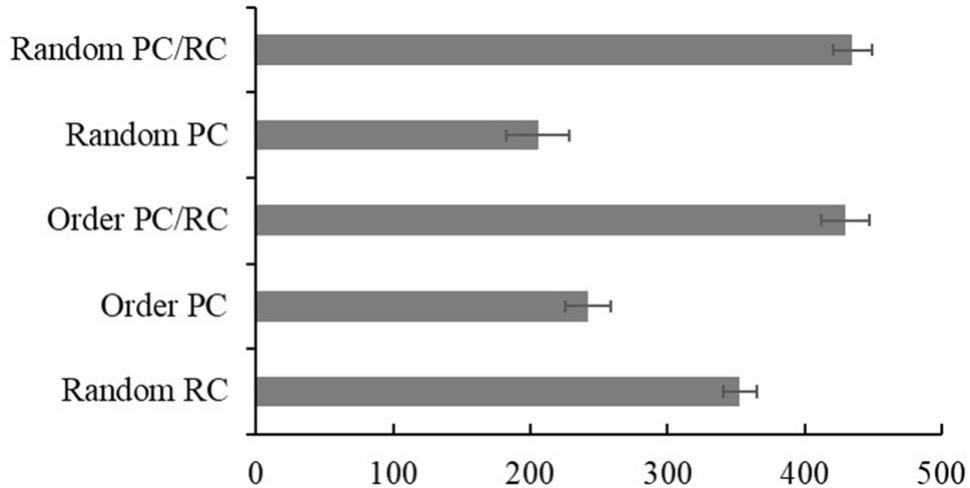
RTs of the switch costs in each condition for Study 2. Error bars indicate standard error

RTs for incorrect responses and trials following incorrect responses were not analysed. We found switch costs for all conditions (Fig. 6). Bonferroni corrected paired samples *t* tests revealed that responses to switch trials [Random PC/RC: *M* = 1187.01, SD 253.83; Order PC/RC: *M* = 1256.18, SD 222.33; Random PC: *M* = 1201.34, SD 314.13; Order PC: *M* = 989.48, SD 278.25; Random: *M* = 1226.58, SD 199.17] were slower than repeat trials [Random PC/RC: *M* = 796.63, SD 145.49; Order PC/RC: *M* = 829.20; SD 178.52; Random PC: *M* = 1008.13, SD 231.93; Order PC: *M* = 763.19, SD 163.34; Random: *M* = 884.62, SD 125.24] in Random PC/RC [*t*(102) = 17.47, *p *< 0.001], Order PC/RC [*t*(104) = 23.95, *p *< 0.001], Random PC [*t*(102) = 10.51, *p *< 0.001], Order PC [*t*(104) = 10.68, *p *< 0.001], Random [*t*(102) = 21.94, *p *< 0.001].

Pearson correlation coefficients revealed significant correlations (Table 3). Inattention was correlated with letter monitoring (*r* = − 0.19, *p* =0.04) and OSPAN (*r* = − 0.29, *p* =0.002) scores. Inattention was also correlated to switch costs in random PC/RC (*r* = 0.20, *p *= 0.04) and order PC/RC (*r* = 0.22, *p *= 0.018) conditions. Composite scores of ADHD were also correlated to OSPAN (*r* = − 0.29, *p* =0.002). Scores of SPM were correlated with letter monitoring (*r* = 0.31, *p* =0.001), task switching in random (*r* = 0.20, *p* =0.038) and order PC (*r* = 0.40, *p* <0.001) conditions.

We used Bayes Factors (B) to assess the strength of evidence in support of hypotheses when the p value for the predictors was not significant. We followed Dienes (2014) to assess the strength of evidence in support of hypotheses when the *p* value for the predictors was not significant. Where a Bayes Factor is given, we modelled the predictions of the theory of some evidence for a relationship with a half-normal whose mean and standard deviation values were taken from the variable inattention in the model.

#### Inattention and goal neglect

We ran multiple regression analysis where inattentive and hyperactive/impulsive traits were used as predictors for letter monitoring performance (Table 4). The model explained 14% of the variation in, *F*(3, 115) = 5.98, *p *< 0.001. SPM and inattention were the significant predictors where hyperactivity/impulsivity was non-significant with Bayes Factors providing evidence for the null (*p* = 0.292, *B*_H(0, 0.636)_ = 0.26). The prior was taken from the variable inattention from the same model.

**Table 4:** Summary of regression model for inattention and hyperactivity/impulsivity scores on letter monitoring scores when controlling for SPM scores (Study 2)

Variable	*b*	SE*b*	*β*	*T*	*R*^2^	Adjusted *R*^2^	Semi-partial correlation
SPM	0.49	0.14	0.30	3.46**	0.14	0.12	0.31
Inattention	− 1.52	0.65	− 0.25	− 2.39*			− 0.22
Hyperactivity/impulsivity	0.63	0.60	0.11	1.06			0.10

*p < 0.05, **p < 0.01

#### Inattention and working memory

We ran multiple regression analysis where inattentive and hyperactive/impulsive traits were used as predictors for Operation Span Task performance (Table 5). The model for random PC/RC explained 10% of the variation in, *F*(3, 106) = 3.81, *p *= 0.012. Inattention was a significant predictor, whereas hyperactivity/impulsivity (*p* = 0.592, *B*_H(0, 1.801)_ = 0.17) and SPM (*p* = 0.609, *B*_H(0, 1.801)_ = 0.04) were non-significant with Bayes Factors providing evidence for the null.

**Table 5:** Summary of regression model for inattention and hyperactivity/impulsivity scores on OSPAN scores when controlling for SPM scores (Study 2)

Variable	*b*	SE*b*	*β*	*T*	*R*^2^	Adjusted *R*^2^	Semi-partial correlation
SPM	0.21	0.41	0.05	3.46	0.10	0.07	0.05
Inattention	− 4.59	1.80	− 0.28	− 2.55*			− 0.24
Hyperactivity/impulsivity	− 0.89	1.66	− 0.06	− 0.54			− 0.05

*p < 0.05, **p < 0.01

#### Inattention and task switching

Due to the significant correlation, we ran multiple regression analysis to investigate the role of ADHD traits when explaining switch costs for the random PC/RC and order PC/RC conditions where the use of both proactive control and reactive control was possible.

The multiple regression analysis revealed that the model explained 6% of the variation, *F*(2, 106) = 3.18, *p* = 0.046 (Table 6). Hyperactivity/Impulsivity (*p* = 0.159, *B*_H(0, 30.03)_ = 0.12) was non-significant with Bayes Factors providing evidence for the null. Thus, inattention was the only predictor of the predictable switch cost.

**Table 6:** Summary of regression model for inattention and hyperactivity/impulsivity scores on random PC/RC condition in Study 2

Variable	*b*	SE*b*	*β*	*T*	*R*^2^	Adjusted *R*^2^	Semi-partial correlation
Inattention	75.70	30.03	0.28	2.52*	0.08	0.05	0.24
Hyperactivity/impulsivity	− 39.98	28.20	− 0.16	− 1.42			− 0.14

*p < 0.05, **p < 0.01

The multiple regression analysis revealed that the model for the order PC/RC switch costs explained 8% of the variation, *F*(3, 110) = 2.92, *p* = 0.037 (Table 7). Hyperactivity/Impulsivity **(***p* = 0.722, *B*_H(0, 34.926)_ = 0.12) and SPM (*p* = 0.110, *B*_H(0, 34.926)_ = 0.14) were non-significant predictors with Bayes Factors providing evidence for the null. Thus, inattention was the only predictor of the predictable switch cost. Since SPM was correlated with switch costs in random PC/RC, it was included in the model as a control variable.

**Table 7:** Summary of regression model for inattention and hyperactivity/impulsivity scores on order PC/RC condition when controlling for SPM scores (Study 2)

Variable	*b*	SE*b*	*β*	*T*	*R*^2^	Adjusted *R*^2^	Semi-partial correlation
SPM	12.25	7.61	0.15	1.61	0.08	0.05	0.15
Inattention	80.41	34.93	0.25	2.30*			0.22
Hyperactivity/impulsivity	− 11.73	32.85	− 0.04	− 0.36			− 0.04

*p < 0.05, **p < 0.01

**Fig. 7:**
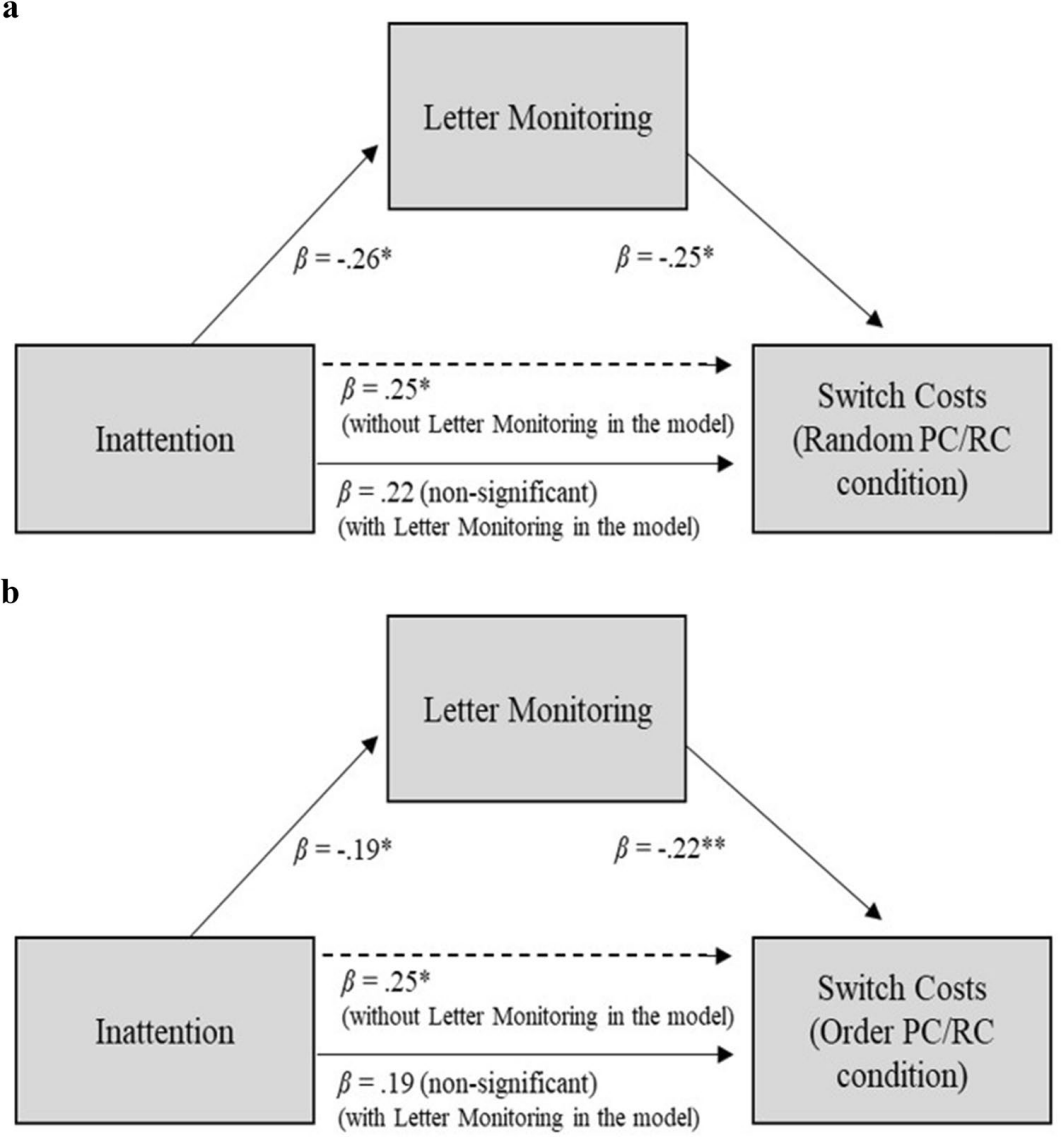
The mediation effect of Letter monitoring performance on the link between inattention scores and switching performance of random (**a**) and order (**b**) PC/RC (Study 2). Bootstrapping was used to calculate a 95% confidence interval around the indirect effect using 1000 resamples

We also ran a mediation analysis using PROCESS Version 3.0 (Hayes, 2018), to investigate a potential mediating role for letter monitoring performance on the relationship of inattention scores with switch costs on random PC/RC and order PC/RC conditions when controlling for hyperactivity/impulsivity and SPM scores. We found inattention was no longer a significant predictor after accounting for the letter monitoring scores, and, letter monitoring was a significant predictor for random (*β *= − 0.25, *p* = 0.020) and order (*β *= − 0.26, *p* = 0.011) PC/RC conditions. Thus, letter monitoring scores mediated the link between inattention scores and the switch costs on PC/RC conditions when the use of both proactive and reactive control was possible (Fig. 7).

### Discussion

The present study had two aims: (1) to de-confound aspects of the design of Study 1 that rendered it difficult to interpret the relationship reported between predictable task switching and inattention (stimulus valency, the presence of an RSI manipulation design permits the de-confounding of working memory load and proactive control); (2) to investigate whether the relationship between inattention and predictable task switching is moderated by goal neglect. It was noted that if inattention were related to impairment in the use of proactive control it would be related to performance in any block that presents an advanced cue (Random PC/RC or Random PC). Alternatively, if inattention were related to working memory impairments it would be related to performance in any block;/condition that has a predictable sequence and involves the need to keep track of the order of switch and repeat trials (Order PC/RC or Order PC), but especially Order PC where no other cue is provided about which task to perform. Finally, if inattention were related to an impairment in reactive control it would affect performance most clearly in the Random RC block. We also conducted Operation Span Task and Letter Monitoring Task to measure the role of WM and goal neglect on the relationship between inattentive traits and predictable switching performance.

Consistent with previous research (Elisa et al., 2016), inattentive traits were unique predictors of the letter monitoring and OSPAN scores when controlling for hyperactive/impulsive traits. However, the results from the order PC and random PC blocks did not replicate those of Study 1. A replication of the results from Study 1 would have been represented by a relationship between inattention and the Order PC condition. This was not observed. Given we observed no relationship between inattention and the Order PC, Random PC and Random RC blocks and inattention (or any of the other ADHD symptoms) our data suggest that those high in inattention do not experience difficulties with keeping tracking of task order, the use of proactive control or the use of reactive control, respectively. The Order PC/RC and Random PC/RC conditions, which were related to inattention, differed from all other conditions by including the possibility of employing both types of control on each trial. Our findings showing that the impairments in switching were mediated by the tendency for goal neglect permit us to conclude that it is likely that inattentive participants were neglecting proactive control and relying on reactive control to complete the task.

The relationship between inattentive traits and switch costs in random PC/RC and order PC/RC is consistent with the literature reporting that individuals with low WM capacity use proactive control less compared to those with high WM capacity (Engle and Kane, 2003; Redick et al., 2011; Unsworth et al., 2004). Given the negative relationship between inattentive traits and measures of WM in the current study (OSPAN scores) and in the literature (e.g. Gathercole et al., 2008; Lui and Tannock, 2007), it is reasonable to expect the decreased use of proactive control as the scores of inattentive traits increase. However, it is important to note that OSPAN scores were not correlated to the switch costs in any of the blocks. Thus, the present findings stress the role of goal neglect in the failure to appropriately weight all aspects of task instructions in the task model, rather than the OSPAN performance, involving maintenance plus manipulation of information on the trial in task switching performance.

Consistent with Verbruggen et al. (2007) who reported successfully eliminated switch costs when use of the advanced cue was strongly encouraged, the presence of an advanced cue or trackable order in the absence of any other cue appears to have encouraged those high in inattention to prepare in advance. In the random PC and order PC conditions, the screen only contained a black frame and a digit which could indicate either of the tasks. Therefore, the only way to know the next task was to keep track of the task order (in order PC) or focusing on the advanced cue (in random PC). Therefore, the goal of attending the cue was reinforced in these conditions.

It is unclear why the Order PC condition did not replicate the high WM condition of Study 1. However, a key difference between the two conditions was the presence of an empty black square 250 ms prior to the onset of the stimulus in Study 2. It is possible that this square served as a cue to withdraw the previous event from memory and remind them of the need to prepare in time for the upcoming trial; we consider this especially likely given that in other conditions the square could be informative. That is, the pseudo-cue in Study 2 acts as a nudge to prevent goal neglect. There is some precedence for this in the goal neglect literature. Duncan et al. (1996) noted that verbal prompts were enough to prevent the occurrence of goal neglect in the letter-monitoring task. Likewise, Parris et al. (2012) also used goal-related primes to prompt the goal of responding quickly and accurately during Stroop performance which resulted in the elimination of Stroop interference. These studies suggest that it is possible that a stimulus that has previously acted as a cue to prepare for an upcoming trial might serve as a reminder of the need to prepare, even if that cue was not being utilised efficiently when it was informative.

## General discussion

In two studies, we conducted predictable and unpredictable task switching paradigms (TSPs) to investigate the link between inattentive traits and task switching performance. Study one revealed that inattentive traits uniquely predicted higher switch costs when there was a set task order that needed to be tracked to permit preparatory control. Study 2 revealed that it was not the ability to perform preparatory processes per se that led to the association between switch costs and inattention, but instead, it was the tendency for those high in inattention to neglect preparatory processes, especially when reactive control options were available. Importantly, the task switching impairment in those high in inattention was related to performance on a goal neglect task. This indicates that the lack of preparatory control was related to a newly reported capacity limit reported by Duncan et al. (1996, 2008) that they have linked to the episodic buffer component of working memory. Another way to understand the present results are as a failure to engage in preparatory control despite the capacity to do so (De Jong, Berendsen, & Cools, 1999). The finding that task switching performance is linked to the tendency for goal neglect in only inattentive participants is consistent with previous work showing that goal neglect is unique to inattention (Elisa et al., 2016).

Consistent with the mixed findings on the relationship between ADHD and task switching performance reviewed in the introduction, our composite scores of ADHD tendencies (CAARS-S:S -index in study one and ASRS-total in study two) were not related to switch costs. It is interesting that inattentive tendencies alone were related to predictable switch costs whilst composite tendencies of ADHD were not. Such a finding highlights the importance of considering the role of individual symptoms when investigating ADHD, at least at subclinical levels. Indeed, the idea of measuring ADHD as a continuum has been proposed. The idea is that a clinical diagnosis represents the extreme end of the inattention, hyperactivity and impulsivity continuums (Barkley and Murphy, 1998). Our findings are consistent with previous studies measuring sub-clinical ADHD traits on a continuous scale (Seli et al., 2015; Elisa et al., 2016; Overbey et al., 2011; Lui and Tannock, 2007) and as with those studies, have implications for clinical level inattention.

Consistent with other studies in the literature (Gathercole et al., 2008; Lui and Tannock, 2007) our results showed that OSPAN scores do not correlate with switch costs (see also Kane et al., 2007; Logan, 2004 for no relationship between WM measures and task switching performance) and is thus supportive of the notion that working memory is independent of task switching capacity (Miyake, Friedman, Emerson, Witzki, Howerter, & Wager, 2000). However, it has been argued that goal neglect is related to an impairment in the episodic buffer of Baddeley’s (2000) working memory model (Duncan et al., 2008) indicating that there might be aspects of working memory that are related to task switching performance.

In summary, in two studies, we measured the link between task switching performance and self-reported ADHD traits. In both studies we report increased switch costs in those high in self-reported inattention. We have concluded that the increased switch costs are due to the frequent failure to engage in preparatory proactive control, especially when the ability to use reactive control is available. The mediation of the impairment in the use of proactive control by goal neglect indicates that the proactive component of the instructions was under-weighted as part of the task goal.
